# Early Single-Center Experience with the Ascyrus Medical Dissection Stent in Acute Type A Aortic Dissection Repair

**DOI:** 10.1055/a-2934-2828

**Published:** 2026-08-19

**Authors:** Raymond Shi, Mathew Doyle, Jada Ching, Bruce French

**Affiliations:** 1 Department of Cardiothoracic Surgery 34378 Liverpool HospitalSydneyNew South WalesAustralia; 2 Department of AnalyticsChing Data AnalyticsSydneyNew South WalesAustralia

**Keywords:** aortic dissection, hemiarch, AMDS

## Abstract

**Background:**

Acute Type A aortic dissection (ATAAD) is a cardiac surgical emergency associated with high rates of mortality and morbidity. The Ascyrus Medical Dissection Stent (AMDS) is a novel hybrid device that has demonstrated promise in stabilizing the aortic arch, eliminating distal anastomosis new reentry tears and improving malperfusion syndromes. This report examines early outcomes of ATAAD patients treated with the AMDS device compared with a sole hemiarch repair.

**Methods:**

This retrospective study included all patients who underwent emergency surgery for computed tomography confirmed ATAAD at our center from January 2024 to November 2025. Iatrogenic, subacute, and chronic dissections were excluded from the study as were patients who received any form of arch surgery.

**Results:**

Six patients received the AMDS device and hemiarch, whereas 18 patients received a hemiarch repair only. A 30-day mortality was 16.7 and 22.2% in the AMDS and hemiarch cohorts, respectively ( *p* >0.99). The 30-day stroke rate was 50 and 27.8% in the AMDS and hemiarch cohorts, respectively ( *p* =0.36). Mean intensive care unit length of stay was 11 days in both cohorts, whereas hospital length of stay was 20 and 18 days in the AMDS and hemiarch cohorts, respectively.

**Conclusion:**

This study has demonstrated the relative safety of the AMDS device when used as an adjunct to the standard hemiarch repair in the management of ATAAD patients. Long-term outcome data are required to determine the extent of the positive aortic remodeling effect of the AMDS device and its impact on aortic reintervention and survival.

## Introduction

Acute Type A aortic dissection (ATAAD) is a cardiac surgical emergency associated with high rates of mortality and morbidity. Perioperative mortality rates of 17–25% have been quoted in literature, despite the substantial improvements attributed to modern surgical and anesthetic techniques. 1​ 2​ ​3​ Currently, in ATAAD patients without an intimal tear in the arch or extensive arch aneurysm, both American and European guidelines recommend the standard hemiarch repair over an extended aortic arch replacement. 2​ ​3​ A hemiarch repair effectively eliminates the primary entry tear in the ascending aorta but often leaves the residual false lumen (FL) in the aortic arch or distal aorta untreated. The residual FL increases the risk of distal anastomotic new entry tear (DANE) formation, and with ongoing pressurization and pulsatile antegrade flow in the FL, prevents true lumen (TL) reexpansion and negatively impacts aortic remodeling. 4​ ​5​ The incidence of DANE after hemiarch repair has been reported to be as high as 70% and is a demonstrated independent risk factor for late distal aortic enlargement requiring reintervention. 6​ 7​ ​8​ Furthermore, a patent FL has been demonstrated to decrease survival and increase distal aortic events. 9​ The Ascyrus Medical Dissection Stent (AMDS) is a hybrid device composed of a proximal polytetrafluoroethylene sewing cuff with a distal uncovered self-expanding nitinol stent designed to exert a chronic low outward force that stabilizes the TL without injuring the friable dissected aortic tissues. Recent studies have demonstrated much promise in the ability of the AMDS device in reducing malperfusion through positive aortic remodeling, achieved by eliminating DANE. 4​ 10​​ 11​ ​12​ This report examines the early outcomes of ATAAD patients treated with the AMDS device as an adjunct to the hemiarch repair compared with a hemiarch repair alone.

## Materials and Methods

### Eligibility Criteria

All patients who underwent emergency surgery for computed tomography (CT) confirmed ATAAD at our center from January 2024 to November 2025 were included. Based on the individual surgeon’s pre- and intraoperative assessment, patients received either an AMDS device and hemiarch repair or a hemiarch repair without the device. No included patients received any form of arch surgery including supra-aortic vessel debranching. Iatrogenic, subacute and chronic dissections were excluded from the study as were patients who had an arch or proximal descending thoracic aortic tear either demonstrated on CT or found intraoperatively. Patients with known Genetic aortopathy (e.g., Marfan’s syndrome) or nickel allergy were excluded due to being contraindicated for AMDS implantation. 11​ ​13​

### Definitions and Outcomes

Preoperative malperfusion was defined as a loss of adequate arterial blood supply due to the dissection and subdivided into coronary, cerebral, peripheral, and visceral categories. Malperfusion was deemed present if either clinical or radiological evidence was demonstrated. Radiological malperfusion was verified by significant compression or complete occlusion by the FL, hence interrupting arterial flow to the relevant organ system. The primary endpoint 30-day mortality was defined as all-cause mortality within the first 30 days after surgery. Secondary endpoints were serious adverse events associated with surgery and include reintubation, dialysis, stroke, revision for postoperative bleeding, postoperative mechanical support, and AMDS complications. AMDS complications were assessed based on postoperative CT imaging performed within 30 days of surgery. Postoperative mechanical support refers to either the use of an intra-aortic balloon pump or extracorporeal membrane oxygenation.

### Statistical Analysis

Continuous variables were described as means with standard deviations and categoric variables were described as total numbers with percentages of the cohorts they represented. Statistical analysis was performed with Stata/MP (version 13.0 StataCorp). Given the small and unbalanced cohorts normal distribution was not assumed. Continuous variables were compared with the two-tailed Mann–Whitney *U* test and categorical variables compared by two-tailed Fisher’s exact test. A *p* -value of <0.05 was considered to be statistically significant.

## Results

During the study period, a total of 34 ATAD patients presented to our center. Six patients received the AMDS device and hemiarch repair, whereas 18 patients received a hemiarch repair only. Ten patients were excluded from the study due to surgery involving supra-aortic vessels.

### Baseline Characteristics

The baseline characteristics for patients in the AMDS and hemiarch cohorts are presented in Table 1 . Mean age was 60 and 63 with female gender representing 33 and 50% of the AMDS and hemiarch cohorts, respectively. No significant difference was found between the AMDS and hemiarch cohorts regarding demographics, comorbidities, or preoperative malperfusion. There was a statistically significant difference in the presence of pericardial effusion with zero patients in the AMDS cohort compared with 55.6% of patients in the hemiarch cohort ( *p* =0.02). No patients in the AMDS cohort presented with tamponade as opposed to 22.2% in the hemiarch cohort ( *p* =0.54). In the hemiarch cohort 16.7% of patients required preoperative intubation and 27.8% of patients required preoperative catecholamine therapy. No patients in the AMDS cohort required preoperative intubation or catecholamine therapy. The AMDS cohort did not have any patients who suffered from chronic renal failure, chronic obstructive pulmonary disease, or had previous cardiac surgery. Preoperative coronary and visceral malperfusion was 50 and 83.3% in the AMDS cohort compared with 22.2 and 44.4% in the hemiarch cohort, respectively. All patients in the AMDS cohort presented with moderate or severe aortic regurgitation compared with 66.7% of patients in the hemiarch cohort.

**Table:** **Table 1** Baseline characteristics

	AMDS	Hemiarch	*p* -value
Age (y)	60 (9)	63 (13)	0.55
Gender (female)	2 (33.3)	9 (50)	0.65
BMI (kg/m ^2^ )	31 (9.3)	28.7 (7.1)	0.74
BSA	2.1 (0.2)	1.9 (0.3)	0.29
Comorbidities			
Hypertension	3 (50)	10 (55.6)	>0.99
Diabetes	1 (16.7)	1 (5.6)	0.45
Chronic renal failure	0	2 (11.1)	>0.99
Coronary artery disease	0	0	
COPD	0	2 (11.1)	>0.99
Current smoker	1 (16.7)	5 (27.8)	>0.99
Previous cardiac surgery	0	1 (5.6)	>0.99
Previous stroke	0	0	
Presentation			
Acute shock	4 (66.7)	8 (44.4)	0.64
Preoperative intubation	0	3 (16.7)	0.55
Preoperative catecholamine therapy	0	5 (27.8)	0.28
Pericardial effusion	0	10 (55.6)	0.02
Tamponade	0	4 (22.2)	0.54
Malperfusion			
Coronary	3 (50)	4 (22.2)	0.31
Cerebral	2 (33.3)	7 (38.9)	>0.99
Peripheral	2 (33.3)	1 (5.6)	0.14
Visceral	5 (83.3)	8 (44.4)	0.17
Aortic regurgitation			
None or mild	0	6 (33.3)	0.28
Moderate or severe	6 (100)	12 (66.7)	0.28

Abbreviations: BMI, body mass index; BSA, body surface area; COPD, chronic obstructive pulmonary disease.

Note: Values are presented as mean (standard deviation) or *n* (%).

### Operative Data

Operative data are presented in Table 2 . There was no significant difference between the AMDS and hemiarch cohorts with regard to operative duration. The mean circulatory arrest duration was 48 min in the AMDS cohort compared with 43 min in the hemiarch cohort ( *p* =0.34). The right axillary artery was selected as the arterial cannulation site for cardiopulmonary bypass (CPB) in 33% of patients in the AMDS cohort compared with 72.2% in the hemiarch cohort ( *p* =0.15). The femoral artery was used in 66.7% of patients in the AMDS cohort compared with 22.2% in the hemiarch cohort ( *p* =0.13). There was a statistically significant difference in the route of antegrade cerebral perfusion (ACP). ACP was given via the right axillary artery in 33.3% of patients in the AMDS cohort compared with 77.8% in the hemiarch cohort ( *p* =0.03). A total of 66.7% of patients in the AMDS cohort received an aortic root replacement compared with 11.1% in the hemiarch cohort ( *p* =0.02).

**Table:** **Table 2** Operative data

	AMDS	Hemiarch	*p* -value
Operation duration (min)	425 (90)	436 (109)	0.66
CPB duration (min)	281 (87)	259 (92)	0.59
Aortic cross-clamp duration (min)	196 (60)	171 (65)	0.46
Circulatory arrest duration (min)	48 (11)	43 (11)	0.34
ACP duration (min)	38 (17)	33 (11)	0.65
Hypothermia temperature (°C)	21 (2)	21 (3)	0.78
Arterial cannulation for bypass			
Right axillary artery	2 (33.3)	13 (72.2)	0.15
Ascending aorta	0	2 (11.1)	>0.99
Femoral artery	4 (66.7)	4 (22.2)	0.13
ACP route			
Right axillary artery	2 (33.3)	14 (77.8)	0.03
Left common carotid artery	3 (50)	7 (38.9)	>0.99
Operative procedures			
Aortic rupture	1 (16.7)	1 (5.6)	0.45
Aortic root replacement	4 (66.7)	2 (11.1)	0.02
Aortic valve replacement	4 (66.7)	6 (33.3)	0.19
Aortic valve repair	1 (16.7)	5 (27.8)	>0.99
Coronary artery bypass	2 (33.3)	4 (22.2)	0.62
OCM with DSC	4 (66.7)	14 (77.8)	0.62
Blood transfusion required	5 (83.3)	16 (88.9)	>0.99
Units transfused within 24 h	5 (4)	7 (6)	0.81

Abbreviations: ACP, antegrade cerebral perfusion; AMDS, Ascyrus Medical Dissection Stent; CPB, cardiopulmonary bypass; DSC, delayed sternal closure; OCM, open chest management.

Note: Values are presented as mean (standard deviation) or *n* (%).

### Postoperative Data

The postoperative data are displayed in Table 3 . There was no significant difference between the two cohorts. A 30-day mortality was 16.7 and 22.2% in the AMDS and hemiarch cohorts, respectively ( *p* >0.99). The 30-day stroke rate was 50 and 27.8% in the AMDS and hemiarch cohorts, respectively ( *p* =0.36). Mean intensive care unit (ICU) length of stay was 11 days in both cohorts, whereas hospital length of stay was 20 days in the AMDS cohort compared with 18 days in the hemiarch cohort. No patients in the AMDS cohort required reintubation, postoperative mechanical support, revision for bleeding, or dialysis for acute renal failure (ARF). Postoperative atrial fibrillation (AF) occurred in 66.7 and 38.9% of patients in the AMDS and hemiarch cohorts, respectively ( *p* =0.36).

**Table:** **Table 3** Postoperative data

	AMDS	Hemiarch	*p-* value
30-d mortality	1 (16.7)	4 (22.2)	>0.99
30-d stroke	3 (50)	5 (27.8)	0.36
ICU length of stay (d)	11 (6)	11 (7)	0.95
Hospital length of stay (d)	20 (8)	18 (8)	0.62
Mechanical ventilation duration (d)	6 (5)	4 (4)	0.31
Reintubation	0	1 (5.6)	>0.99
Postoperative mechanical support	0	3 (16.7)	0.55
Revision for bleeding	0	1 (5.6)	>0.99
ARF requiring dialysis	0	7 (38.9)	0.13
New AF	4 (66.7)	7 (38.9)	0.36
Delerium	1 (16.7)	3 (16.7)	>0.99

Abbreviations: AF, atrial fibrillation; AMDS, Ascyrus Medical Dissection Stent; ARF, acute renal failure; ICU, intensive care unit.

Note: Values are presented as *n* (%).

Table 4 demonstrates AMDS implantation data. All patients had successful implantation of the AMDS device. There was one episode of device related reintervention in a patient who was found to have central stent collapse on postoperative CT imaging.

**Table:** **Table 4** Ascyrus medical dissection stent implantation

Successful deployment	6 (100)
Aortic injury associated with device implantation	0
Stent fracture	0
Distal stent-induced new entry tear	0
Device-related reintervention	1 (16.7)

Note: Values are presented as *n* (%).

## Discussion

The hemiarch approach to ATAAD is a life-saving operation that effectively eliminates the primary entry tear in the ascending aorta but often leaves the FL in the aortic arch or distal aorta untreated. Persisting flow within the FL, along with an increased risk of DANE formation negatively impacts aortic remodeling increasing the risk of malperfusion syndromes hence necessitating later aortic reintervention. 6​ 7​ ​8​ An early total arch replacement (TAR) with a frozen elephant trunk (FET) has been advocated as a method of excluding the FL as well as reentry tears in the arch and distal aorta, preventing negative aortic remodeling and reintervention. In recent years, the in-hospital mortality and stroke rates following early TAR have improved considerably, becoming comparable to the standard hemiarch repair in some expert centers. 14​ However, this aggressive approach is time consuming and technically challenging, often requiring specialist aortic teams to achieve comparable short-term outcomes to the hemiarch repair. 15​ These results may not be feasible outside of specialized aortic centers, especially in the acute emergency setting with a critically unwell ATAAD patient. 2​ 5​​ ​16​ Moreover, there is also a reported 1–5% risk of spinal cord ischemia attributed to FET deployment across the spinal arteries. 14​ In the absence of an intimal arch or distal aortic tear the benefit of the early TAR approach is prophylactic in its potential of avoiding late aortic complications and reinterventions necessitated by the residual pressurized FL and negative aortic remodeling. No prospective controlled studies have assessed the associated increased risk of such an approach against the incidence of late aortic complications and aortic reinterventions in the setting of an ATAAD. Hence, outside of specialized aortic centers an individualized patient-centered approach is recommended considering dissection anatomy, surgical expertise, and other patient factors. 2​

The AMDS device aims to bridge this treatment dichotomy by providing a means to address the residual FL in the aortic arch or distal aorta in the index operation without unnecessarily increasing the complexity and risk of surgery. As an adjunct to the hemiarch repair the AMDS device aims to support the TL, eliminate antegrade pulsatile flow in the FL and prevent DANE formation. This allows for positive aortic remodeling and reduced rates of late aortic reintervention. The technique for AMDS device implantation is well described in literature, 5​ ​12​ being relatively straightforward to allow for ease of successful implantation by all cardiac surgeons in the acute emergency setting without significantly prolonging the operative duration. This retrospective study demonstrates our early short-term experience with the utilization of the AMDS device as an adjunct to a hemiarch repair in the management of ATAAD.

There was no significant difference in the postoperative outcomes of the AMDS and hemiarch cohorts attesting to the relative safety of the AMDS device. The 30-day mortality rate of 16.7% in the AMDS cohort, sitting below the hemiarch cohort of 22.2%, is in line with figures published in literature ranging from 11 to 19%. 4​ 10​​ 11​ ​12​ 17​​ 18​ 19​ ​20​ The ICU and hospital length of stay was comparable between the two cohorts, a finding that was also demonstrated in White and colleague’s study. 4​ In our center, there was a low threshold for open chest management (OCM) with delayed sternal closure (DSC), if there were any concerns of coagulopathy, hence the rates of 66.7 and 77.8% in the AMDS and hemiarch cohorts, respectively. This may have led to a prolonged duration of ICU and hospital length of stay compared with literature. In a propensity score-matched analysis Pitts et al. 11​ demonstrated an ICU length of stay of 9 and 6 days with rates of OCM and DSC of 17 and 12% in the AMDS and hemiarch cohorts, respectively ( *p* =0.076).

The 30-day stroke rate of 50% in the AMDS cohort compared with 27.8% in the hemiarch cohort is of concern. This rate is considerably higher than figures reported in literature which range from 2 to 37.5%. 4​ 10​​ 11​ ​12​ 17​​ 19​​ ​20​ The lack of statistical significance ( *p* =0.36) and small sample size makes it difficult to interpret as directly associated with the AMDS device implantation. Preoperative cerebral malperfusion was similar in both cohorts at 33.3 and 38.9% in the AMDS and hemiarch cohorts, respectively. The mean circulatory arrest and ACP duration was slightly prolonged in the AMDS cohort at 48 and 38 min compared with 43 and 33 min in the hemiarch cohort. This reflects the additional time taken for AMDS implantation and is in line with figures published in literature. 4​ 11​​ ​20​ We do not believe that this mean difference of 5 min would contribute significantly to the elevated stroke rate in the AMDS cohort, especially considering the protective effect offered by a mean hypothermic temperature of 21°C in both cohorts. This is reinforced by the Immohr et al. 20​ study that demonstrated a statistically significant difference in the mean circulatory arrest duration (52 min in AMDS and 30 min in control cohorts *p* < 0.01) at a mean hypothermic temperature of 26°C. Although the circulatory arrest time was prolonged due to their preference for an additional suture line and cuffed anastomosis technique in the AMDS cohort they did not experience any increase in stroke rates (22.2% in AMDS and 17% in control cohorts, *p* =0.65).

All patients in the study who developed new AF postoperatively were appropriately anticoagulated according to local hospital guidelines. Thus, it is unlikely that the differing rates of new AF contributed significantly to the 30-day stroke rates between the two cohorts. A meta-analysis by Benedetto et al. 21​ demonstrated reduced risks of in-hospital mortality relative risk (RR) 0.41 (95% confidence interval [CI]: 0.29–0.58; *p* < 0.001) and stroke RR 0.59 (95% CI: 0.37–0.93; *p* =0.02) in ATAAD patients receiving axillary arterial cannulation compared with femoral arterial cannulation (FAC). This effect was hypothesized to be due to retrograde brain embolization, worsening dissection, and organ malperfusion associated with retrograde arterial flow from FAC. 21​ Consequently, the differing rates of 30-day stroke found in our study may be confounded by the preference for FAC representing 66.7% of patients in the AMDS cohort compared with 22.2% in the hemiarch cohort. Selection of arterial cannulation site for CPB in our study was an individual surgeon’s decision based on the patient’s dissection anatomy and hemodynamic status.

Concomitant procedures are known to carry an increased stroke risk compared with isolated procedures. Demal et al. 22​ demonstrated that concomitant coronary artery bypass grafting (CABG) was an independent risk factor for stroke (odds ratio [OR]: 2.19; *p* =0.005) in patients undergoing aortic surgery. In our cohort the rate of concomitant CABG was 33.3 and 22.2% in the AMDS and hemiarch cohorts, respectively. The higher rate of concomitant procedures may also be a contributing factor to the disparate 30-day stroke rates between the two cohorts.

Despite the potential impact of confounding factors, the high 30-day stroke rate in our AMDS cohort remains a notable concern. White et al. 4​ similarly reported a higher stroke rate in the AMDS cohort (21.6%) compared with the hemiarch cohort (14.3%; *p* =0.421), although their study was also limited by small sample size (37 AMDS and 77 hemiarch patients). 4​ In a propensity score matched analysis, Pitts et al. 11​ demonstrated a 4% new stroke rate in both AMDS and hemiarch cohorts, ( *p* =0.99; OR: 0.98, 95% CI: 0.11–8.43). Collectively, the limitations of our study and the potential for multiple confounding factors highlight the need for cautious interpretation of the analysis of our very early institutional experience with the AMDS device as well as the need for further evaluation in larger prospective studies.

No patients in our AMDS cohort developed ARF requiring dialysis compared with a rate of 38.9% in the hemiarch cohort. The reported rates of new ARF requiring dialysis post-AMDS implantation in literature range from 3 to 37%. 4​ 11​​ 17​​ ​18​ ​20​ In a retrospective observational cohort study, Immohr et al. 20​ demonstrated a rate of ARF requiring dialysis of 11.1 and 22.4% in their AMDS and hemiarch cohorts, respectively.

Our AMDS cohort did not have any instances of DANE formation on postoperative CT imaging. This is consistent with findings from both the “dissected aorta repair through stent implantation” (DARTS) trial, 10​ and early results from the “prospective, single-arm, multicenter clinical investigation to evaluate the safety and effectiveness of AMDS in the treatment of acute DeBakey type I dissection” (PERSEVERE) trial. 12​ Pitts et al. 11​ demonstrated a statistically significant reduced incidence of DANE in their AMDS cohort (17%) compared with the hemiarch cohort (45%) based on immediate postoperative CT imaging prior to patient discharge ( *p* =0.028, OR=0.35 [95% CI: 0.13–0.87]). Similarly, White et al. 4​ also demonstrated a statistically significant lower incidence of DANE in their AMDS cohort at 11.8% compared with 43.3% of the hemiarch cohort ( *p* =0.002). Both White et al. 4​ and Pitts et al. 11​ established a statistically significant trend toward TL reexpansion and FL thrombosis in the AMDS cohort as compared with the hemiarch cohort, although there was no statistically significant difference in short-term outcomes.

The benefit of the AMDS device lies in its ability to promote positive aortic remodeling, an effect that will be most evident through analysis of the long-term outcomes. Unfortunately, there remains a paucity of published studies regarding the long-term outcomes post AMDS implantation in ATAAD patients. Recently published 3-year outcomes of the DARTS trial, 10​ demonstrated a partially or completely thrombosed FL of 90.5% in zone 0, 60.0% in zone 1, and 68.2% in zone 2 of the aorta. This remains consistent with the early outcomes published in literature demonstrating a trend for the AMDS device to promote FL thrombosis, particularly in the aortic arch with a reduced effect further downstream. 4​ 10​​ ​11​ Bozso et al. experienced no instances of DANE, and the aortic reintervention rate was 13%, sitting below the aortic reintervention rates posthemiarch repair in ATAAD patients published in literature. 9​ ​10​ 23​​ 24​ ​25​ Upcoming long-term outcome data from the PERSEVERE trial and other studies and registries will help further evaluate the ability of the AMDS device in facilitating positive aortic remodeling and reducing the risks of aortic reintervention and associated mortality and morbidity.

**Fig. 1:**
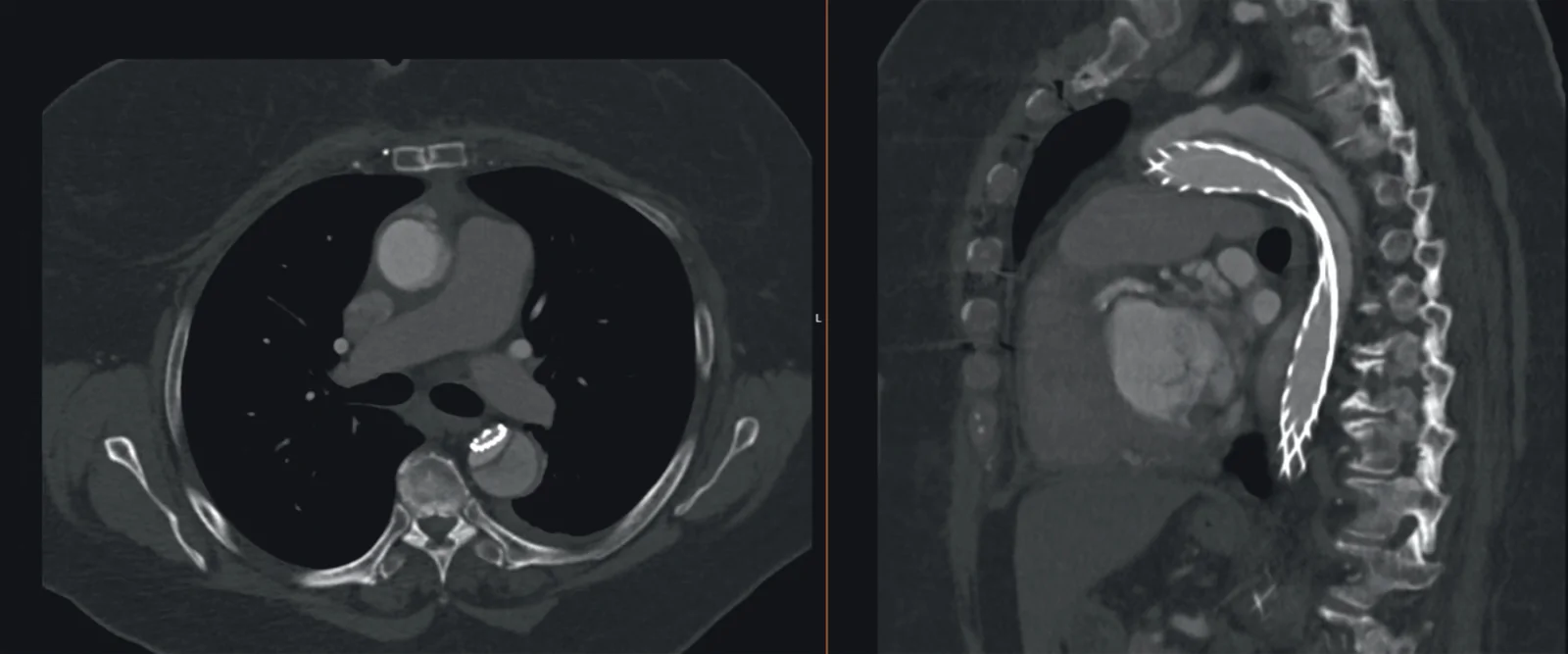
Axial and sagittal CT images demonstrating central stent collapse. CT, computed tomography.

We had one AMDS associated complication in the form of central stent collapse found on routine postoperative CT imaging ( Fig. 1 ). Despite not experiencing any adverse clinical sequalae, we elected to pursue endovascular ballon angioplasty at a later stage to maximize the long-term positive aortic remodeling effects of the AMDS device. Complete expansion of the AMDS device with endovascular ballon angioplasty was later achieved without complications. Luehr et al. 19​ first described central stent collapse after it was observed in 9% of their patients post AMDS device implantation on routine postoperative CT imaging. They hypothesized that this phenomenon was more likely to occur with a combination of conditions including unfavorable anatomy such as gothic arch and aortic kinking; AMDS oversizing or suboptimal aortic transection and increased proximal tension; and aortic elongation during reperfusion. 19​ From a technical standpoint, leaving a longer ascending aortic graft and at least 1 cm of aortic tissue proximal to the take-off of the innominate artery with a transverse aortotomy instead of beveled toward the lesser aortic curvature, as in standard hemiarch repair, will reduce tension toward the sinotubular junction and hence reduce the risk of central stent collapse. 5​ 12​​ 14​​ ​19​

Sizing the AMDS device is based on preoperative CT imaging measurements. The aortic diameter at the level between the innominate and left common carotid arteries (proximal diameter) and at the pulmonary artery bifurcation (distal diameter) are measured. These measurements are then compared with the manufacturer’s sizing chart allowing for selection of an appropriately sized device. Our patient who experienced central stent collapse had a bovine arch with a proximal diameter measuring 36 mm and distal diameter of 30 mm. Hence, the appropriate AMDS device of 55–40 mm was selected based on the manufacturer sizing chart. The proximal diameter measurement of 36-mm sits on the absolute lower limit of the sizing bracket for the 55 mm AMDS device, leaving very little room for error in sizing up the device. Where such measurements lie in the upper or lower limits of the manufacturer sizing charts, we would advocate for extra care and caution in ensuring accurate measurements and mitigating the other conditions that increase the risk of central stent collapse as identified by Luehr et al. 19​

This study is limited by its retrospective nonrandomized nature and small sample size. These constraints increase the risk of selection bias and confounding, and hence, the comparative statistical analysis should be interpreted with caution, as it reflects only the very early stage of our institutional experience with the AMDS device. Additional recruitment and continued follow-up are recommended to assess the long-term outcomes and positive aortic remodeling effect of the AMDS device in ATAAD patients.

## Conclusion

This study has demonstrated the relative safety of the AMDS device when used as an adjunct to standard hemiarch repair in the management of ATAAD patients. Further large-scale randomized control studies are required to determine if any increase in stroke risk exists with the AMDS device implantation. This has not been demonstrated in current literature supporting the safety of the AMDS device. Long-term outcome data are required to determine the extent of the positive aortic remodeling effect of the AMDS device and its impact on aortic reintervention and survival.
